# From digital to physical model: testing of a workflow for case-specific 3D-printed bone simulants

**DOI:** 10.1007/s00414-026-03945-4

**Published:** 2026-08-18

**Authors:** Manon Wartel, Daan Wintermans, Stefan Schaufelbühl, Wim Kerkhoff, Pia Genet, Fabiano Riva

**Affiliations:** 1 Ecole des sciences criminelles, Université de Lausanne, Lausanne, Switzerland; 2 Kompetenzzentrum Forensik, Kantonspolizei St.Gallen, St.Gallen, Switzerland; 3 Netherlands Forensic Institute (NFI), PO Box 24044, The Hague, 2490 AA The Netherlands; 4 University Centre of Legal Medicine Lausanne-Geneva, Lausanne University Hospital and University of Lausanne, Lausanne, Switzerland

**Keywords:** Wound ballistics, Synthetic bone substitutes, Ballistic testing, 3D printing, Femurs, Scapulas, Cortical bone, Cancellous bone, Bone marrow, PLA, TPU, PA6-CF

## Abstract

Bone simulants are used for a variety of purposes in wound ballistic experimentation. Synthetic simulants have several advantages over biological (either human or animal) material. Generic synthetic simulants are available, but the shapes and sizes of these materials cannot cover the full range observed in human individuals. This is a disadvantage, e.g., in forensic reconstructions where accurate and case-specific simulants are often desirable. One way to circumvent this problem is to print bone simulants, based on scanned victims’ data. The current study is intended to explore the feasibility of this method, find suitable materials, and determine optimal printer settings. For this purpose, two porcine scapulas and two porcine femurs were scanned with a Computed Tomography (CT) scanner and replicated with a 3D printer based on the scan data. Polylactic acid (PLA), thermoplastic polyurethane (TPU), and nylon-carbon fibre (PA6-CF) were used as printing materials. Cancellous bone was replicated by printing a honeycomb pattern with a 60% infill. Cortical bone was replicated by printing a rectilinear pattern with a 100% infill. Both the biological originals and their printed replicas were embedded in gelatine and shot with 6,35 mm Browning and .22 Long Rifle bullets. Penetration capacity, projectile deformation and fracture characteristics were compared. The results were most promising with the TPU prototypes. For the PLA and PA6-CF, the fracture characteristics and projectile deformation differed from those observed with the biological originals. Also, the penetration depth into the gelatine behind the bone simulants was lower with these materials.

## Introduction

 The physical injuries caused by firearms are the result of the interaction between a projectile and biological tissues. The field that studies this interaction is called wound ballistics. The knowledge generated by this field can be used for various purposes, e.g., in a forensic context. This knowledge is often still acquired empirically, as it depends on numerous parameters and it remains difficult to model mathematically [1]. Various tissue simulants can be used for experimentation. Synthetic simulants are preferable to biological simulants (from humans or animals) for improved reproducibility and consistent composition, as well as for ethical reasons [2, 3]. Also, biological tissues require specific storage conditions, unlike synthetic simulants.

Several tissue simulants are available and applied in forensic science. With regard to bone simulants, e.g., Synbone^®^ produces polyurethane generic forms, such as cylinders and spheres, that represent long bones and skulls respectively. The literature presents conflicting results regarding Synbone^®^’s ability to accurately reproduce bone behaviour during ballistic impact. Multiple studies indicate a need for further research to develop a substitute that represent human bone better [2, 4–11].

Because human bone structure is complex, simplified models may fail when used to simulate bullet interactions. There are two types of bone tissue: Cancellous (or trabecular) bone forms the inner part of bone and is composed of interconnected studs and plates in an almost honeycomb-like structure. Cortical bone is solid and is stiffer and denser than cancellous bone [12, 13]. Cancellous and cortical bone proportions vary substantially across bones. The diaphysis of long bones (e.g., femurs) consists almost entirely of cortical bone, while flat bones (e.g., scapulas) consist of cancellous bone between two thin layers of cortical bone. Bones also contain bone marrow, a soft tissue essential for blood cell production and tissue renewal. In the diaphysis, bone marrow fills the medullary cavity, whereas in other bones it fills the spaces between the trabeculae of cancellous bone. The average bone marrow density is similar to that of blood or adipose tissues [14].

Generic synthetic bone simulants currently do not fully simulate the intricate structure of biological tissue, nor do they account for the variation in shape and size found in human bones. Using synthetic 3D-printed simulants based on human body scans circumvents these problems. Some ballistic tests on 3D-printed models have already been conducted by Riva et al. [15]. The authors printed 6 mm thick plates from (among other materials) Polylactic Acid (PLA) and Thermoplastic Polyurethane (TPU), and compared their performance with Synbone^®^ plates of the same size. The authors went on to print head models based on scans from deceased shooting incident victims and compared test results on these models to documented aspects of the shooting incidents. In these two experiments, the results showed a better similarity with the TPU simulants in terms of projectile trajectories and wound characteristics.

The objective of the current study is to further develop the production of 3D printed bone simulants. The printable material should be capable of reproducing bullet retardation as well as bullet deformation after impact with bone. Porcine bones are used as a reference because of their similarity to human bone under fracture stress [16]. The synthetic material was printed to the same shape and size as the porcine bones used as a reference. The following steps were taken: (1) create virtual 3D models from the reference bones with a Computed Tomography (CT) scanner; (2) create synthetic replicas in various commercially available materials using 3D printing technology based on these scans; (3) submit the bones and their printed replicas to ballistic loading under identical conditions.

## Materials and methods

### Selected simulants

The same materials were selected that were used earlier by Riva et al. [15]. Because the conclusions in [15] reported less projectile deformation with the tested synthetic materials than with human bone, an additional (stronger) material was added: polyamide reinforced with carbon fibres (PA6-CF).

As bone is always surrounded by soft tissues in living humans and animals and because these soft tissues have an influence on bullet behaviour, both porcine bones and printed simulants were embedded in ballistic gelatine. The gelatine (10%, cooled to 4 °C) was prepared, calibrated and verified according to the guidelines provided by Jussila [17]. See Table 1 for the densities of the various biological and synthetic materials.

**Table 1:** Density values of the different types of biological bones as well as density values and brand of the materials used to reproduce 3D printed replicas

Materials	Type and brand	Density [g/cm³]
Biological cortical bones	/	1.91
Cancellous biological bones	/	1.18
Bone marrow (yellow)	/	0.92
PLA	PLA NX2, Extrudr	1.24
TPU	Flex Hard, Extrudr	1.22
PA6-CF	PA6-CF, Bambu	1.09
Gelatine	Gelita, Ballistic 3	1.03

### Biological originals

**Fig. 1:**
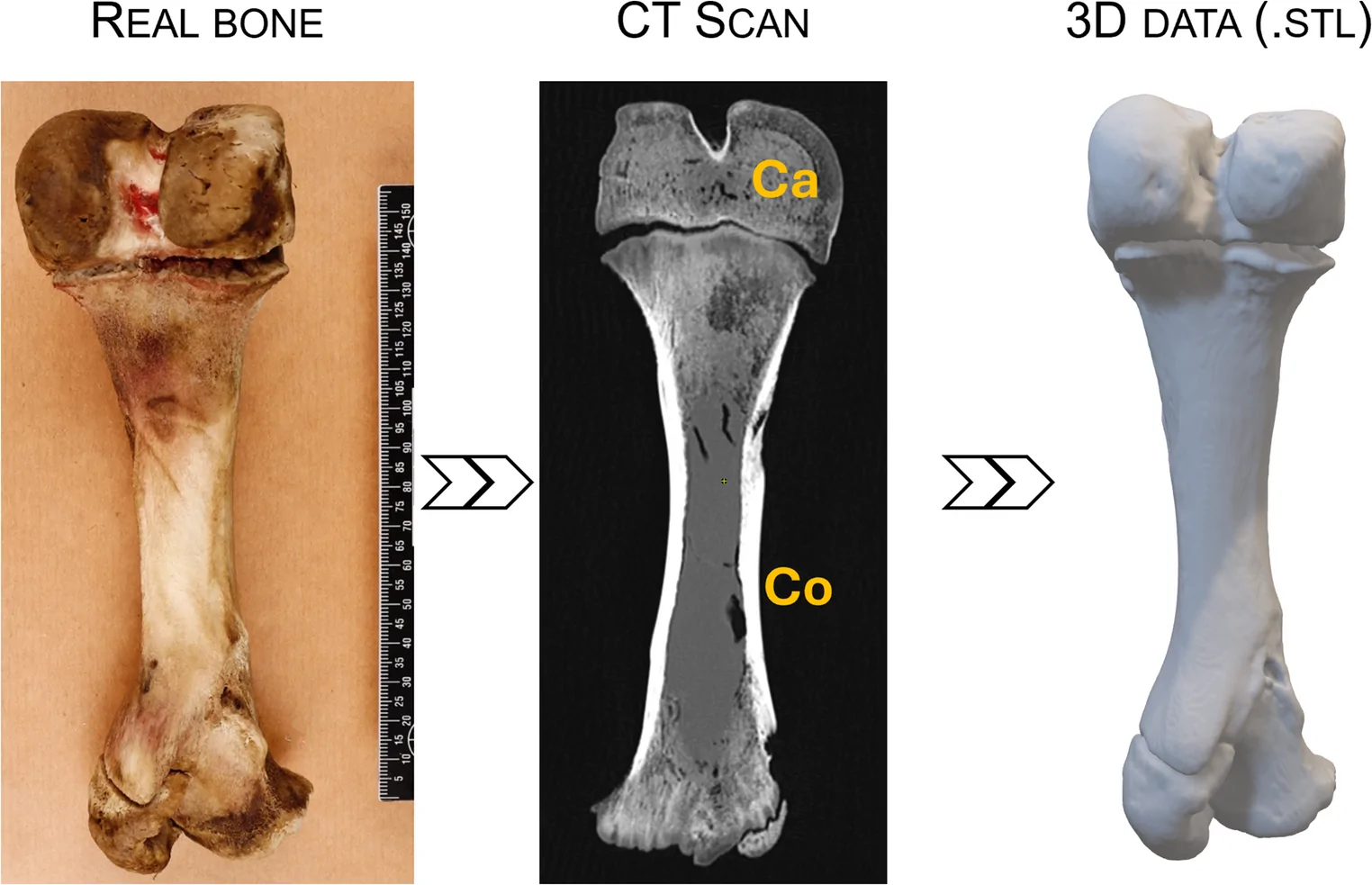
Illustration of the CT scan-based procedure used to create a 3D model (.stl). Left: image of the biological bone. Middle: CT scan image of the biological bone (the letters Ca and Co on the image indicate the cancellous and the cortical bone portion, respectively). Right: the 3D model relative to the outer bone surface extrapolated from the CT data

Two porcine femurs (named A and B) and two porcine scapulas (named C and D) were obtained from a slaughterhouse and macerated to remove residual soft tissue. The bones were treated with a formalin-based fixative for preservation and stored in a freezer at -24 °C (-11 °F) until used. They were then scanned using a GE Healthcare Milwaukee, USA, Discovery HD750 CT-scanner. For each bone, the exterior surface was isolated on the CT-scan images based on its density and exported as .stl file format using the AW Server software (Fig. 1). The resulting 3D models were then “cleaned” in MeshLab software by deleting small artefacts and slightly smoothing surfaces (right image in Fig. 1).

The CT scan images have also been used to measure the thickness of the cortical layer (the white layer indicated by the abbreviation Co in Fig. 1) in relation to the cancellous bone (indicated by the abbreviation Ca in Fig. 1). The thickness measurements have been focused on the intended target area, which is roughly located at the centre of the diaphysis and on a relatively even part of the scapulas. To ensure the shots were placed in the same location on the biological originals and the 3D-printed replicas, the intended impact points were marked. This was done with a marker pen on the porcine bone and with a printed marker in the 3D files on the synthetic replicas.

### 3D files

**Fig. 2:**
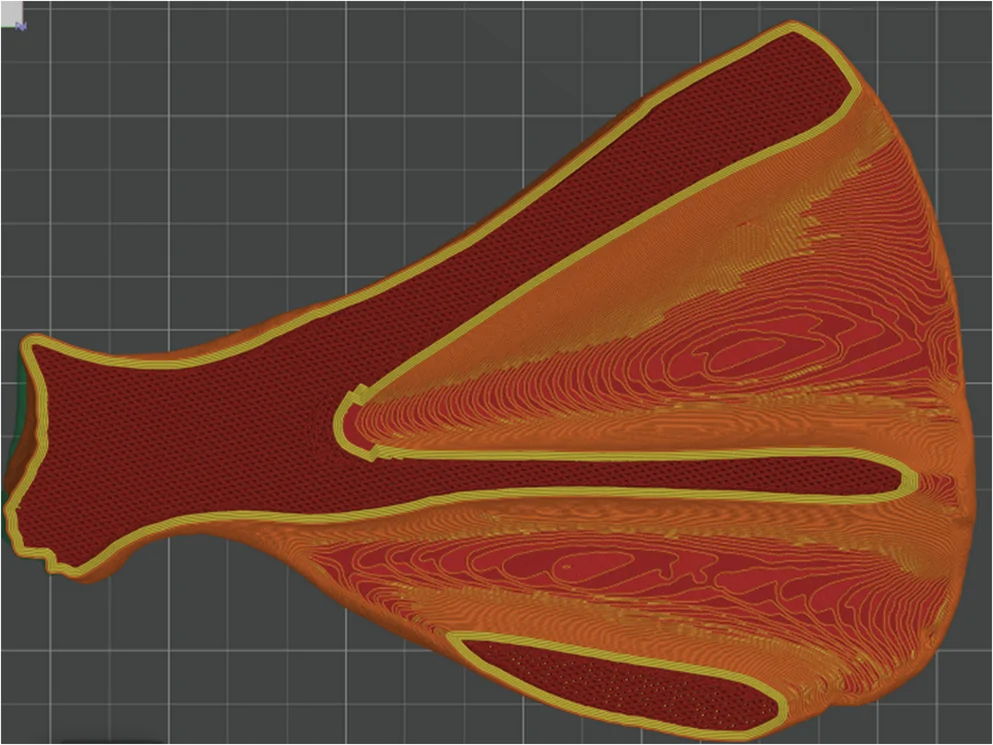
Cross-sectional view of the printing plan for scapula C. The outer yellow layers correspond to the cortical bone portion, while the internal red structure with a filling of 60% should replicate the cancellous bone

The files in .stl format, resulting from the above-mentioned procedure, contain only the outer surface information, without any parameters related to the thickness of cortical walls or the filling density of the cancellous part. To include these parameters in the final replica, this information is embedded in the 3D print files (G-code packed into a .3mf file readable by the printer) with the slicer software. This software defines how the printer will build up the replica. This approach goes beyond reproducing external anatomy but also aims to mimic the internal architecture of the bone. The following printing parameters were chosen:

- The simulated *cortical bone* was created with the number of layers (0.4–0.6 mm for each layer in function of the 3D printer nozzle size) needed to print the thickness measured on the CT scan data (Co in Fig. 1). The cortical part was printed with a density of 100% (yellow outer layer in Fig. 2).
- The simulated *cancellous bone* (Ca in Fig. 1) was created by selecting an infill density of 60% (red internal structure in Fig. 2). This value has been chosen because cancellous bone has an approximate density of 60% relative to cortical bone [18] (see Table 1). A honeycomb infill pattern was selected [13, 19].

**Fig. 3:**
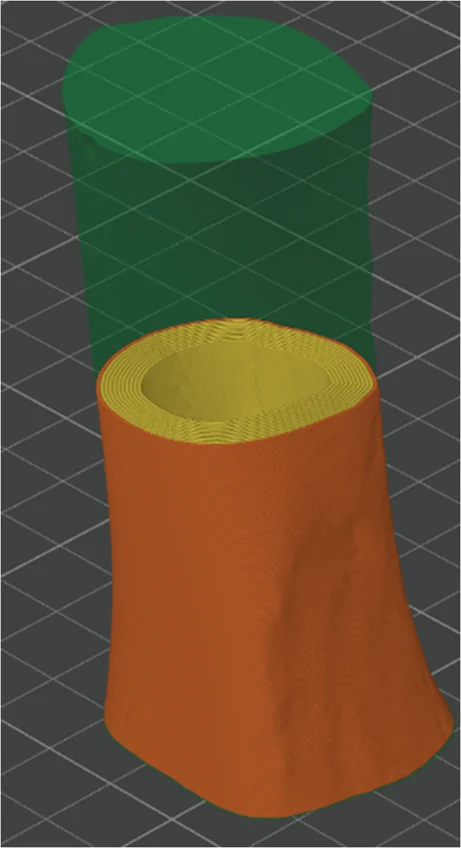
Cross-sectional view at the target level of femur A. The orange and the yellow portions illustrate the outer surface and the cortical bone, respectively. The green semi-transparent part represents the upper part of the femur

The middle part of the simulated femurs (diaphysis) was left hollow and later filled with 10% gelatine as a simulant for bone marrow. The density (Table 1) and mechanical behaviour of bone marrow are reported to be close to that of adipose tissue [14]. See Figs. 2 and 3 for the printing plan for a scapula and (the middle part of) a femur, respectively.

**Fig. 4:**
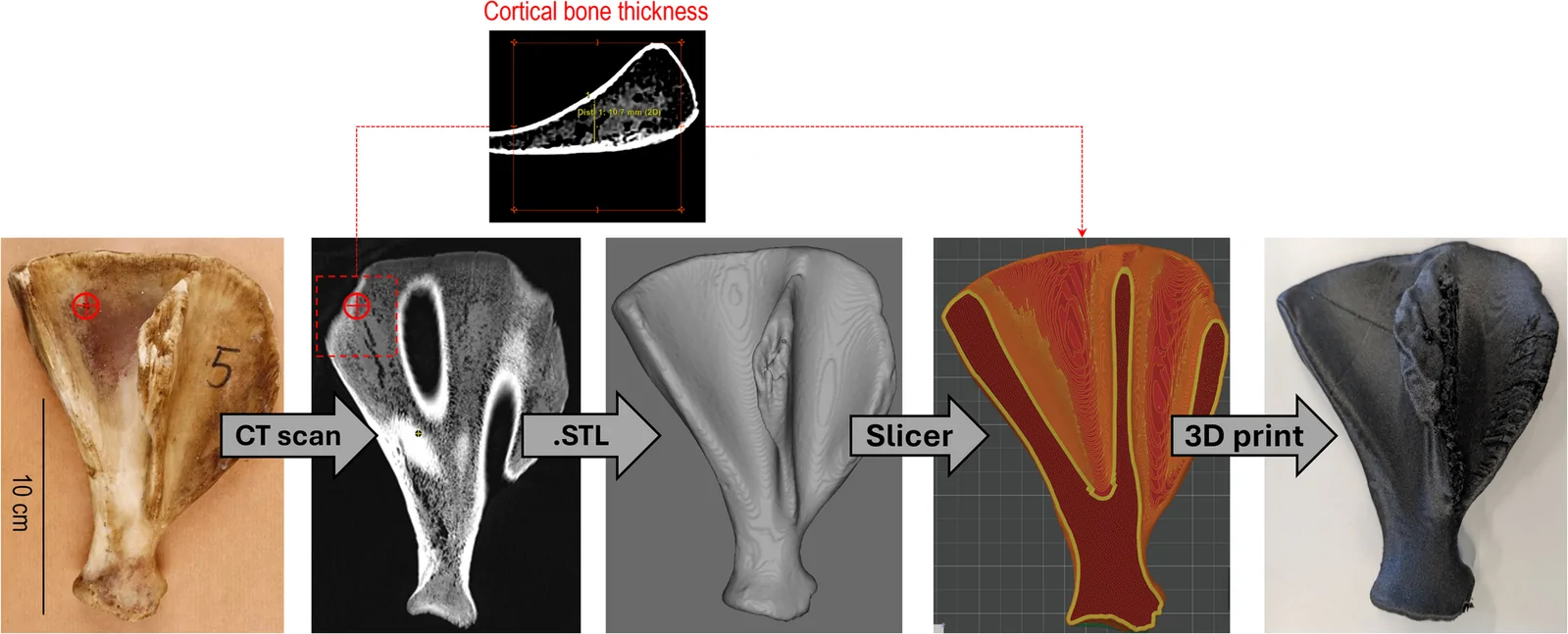
Illustration of the different steps. The biological bone is first scanned with a CT scanner. The resulting CT data are used to measure the cortical bone thickness at the point of impact (marked with the red cross in the circle). The outer bone surface is extracted from the data as an .stl file. This file is imported into the slicer to include bone structure parameters into the final G-code used to print the replica

The whole procedure is illustrated in Fig. 4.

Each sample is printed in three different materials: PLA, TPU and PA6-CF. As this is a pilot study, only one specimen per material and condition was produced and tested (*n* = 1). The results should therefore be interpreted as exploratory and used primarily to guide further testing.

### Ballistics tests

#### Shooting protocol

**Fig. 5:**
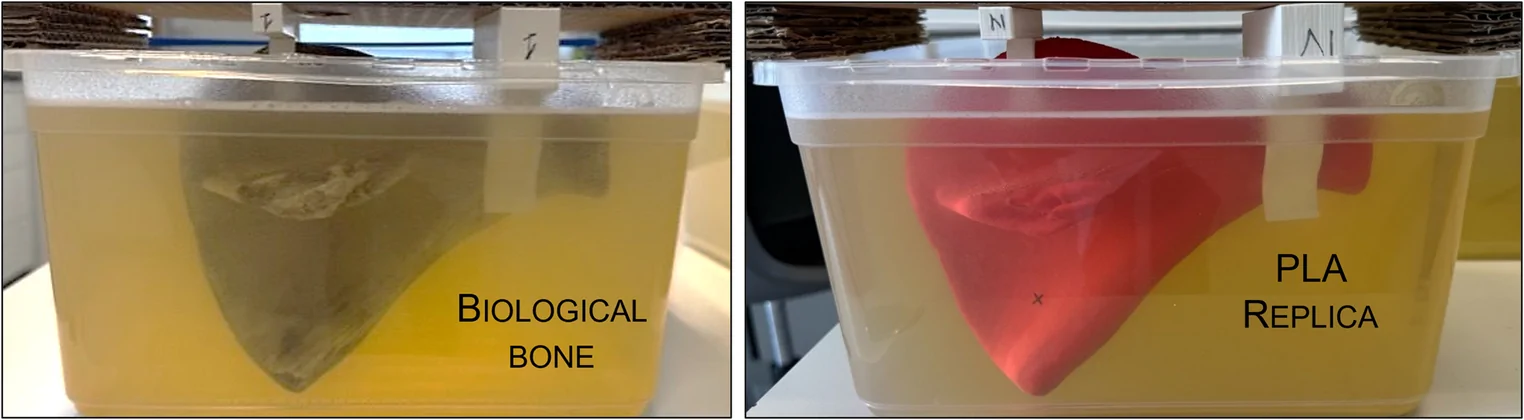
Image showing the phase where the bone samples (biological and 3D printed replica) were immersed in the molten gelatine

The bones and their printed replicas were fixed with clips in a plastic container with a 4 cm distance between the target area and the container edge. The same clips were used for all samples. The liquid (molten) gelatine was poured into the container until the sample was submerged (Fig. 5). The container was then placed in the refrigerator set at 4 °C for at least two days before verifying the conformity of the gelatine by using 4.4 mm steel spheres (Swiss Arms High density Airgun Steel BB’s) according to the instructions given by [17]. For long bones (femurs), because of the size difference between the biological bone and the respective replicas (without epiphysis), the first was placed horizontally into the gelatine, whereas the replicas were placed vertically.

Two different ammunition types were selected. The first is a 6,35 mm Browning Geco brand cartridge with a 3.2 g Full Metal Jacket bullet. This ammunition was fired with an Astra Cub pistol with a 5 cm (2”) long barrel. The second one is a Remington brand .22 Long Rifle cartridge with a 2.6 g lead round nose projectile (brass-coated). This ammunition was fired with a Llama XI revolver with a 10 cm (4”) long barrel. One set (biological and simulated) of scapulas and one set of femurs were shot with 6,35 mm Browning ammunition, and one set of scapulas and femurs was shot with .22 LR ammunition.

**Fig. 6:**
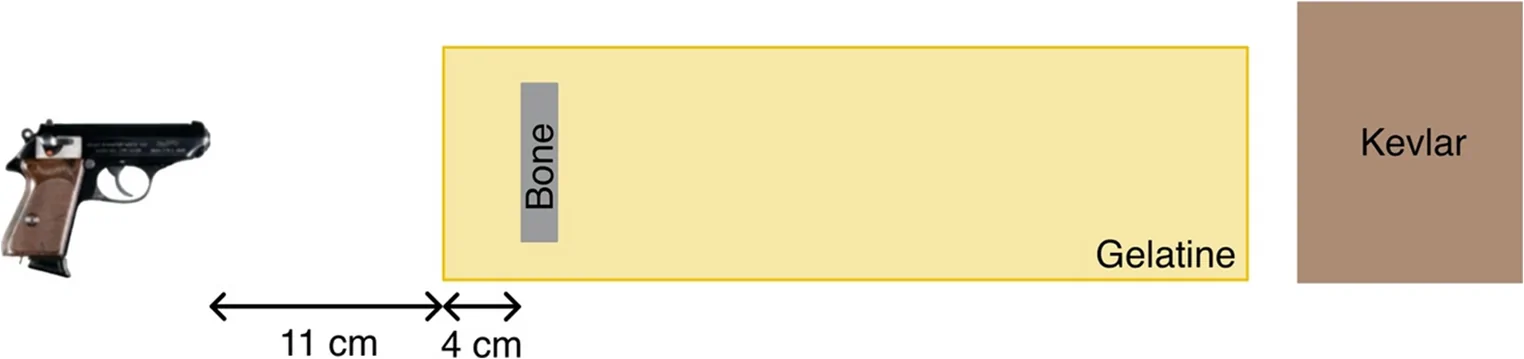
Ballistic test setup used for both ammunition types. A carton box filled with Kevlar was placed behind the gelatine/bone model in case the bullet perforates it completely

The firearms were mounted in a Ranson Rest^®^ with their muzzles at a fixed distance of 11 cm to the front of the gelatine/bone target. A Kevlar-filled box was placed behind the gelatine block to catch the bullet, should it completely perforate the target (Fig. 6).

The 11 cm distance was chosen to ensure high precision during the shooting tests. This short distance did not allow velocity detection. For this reason, test shots were fired beforehand to measure the mean projectiles velocity of the respective weapons. The mean velocity of the 6,35 mm Browning bullets was measured to be 221,8 ± 3,1 m/s, whereas the velocity of the .22 LR bullets was measured to be 311,2 ± 19 m/s.

#### Studied features

For each shot, three features are investigated: (1) the penetration depth of the projectile, which is indicative of the energy loss after impact with the bone and its replicas; (2) the bullet deformation, which provides information about the interaction between the projectile and the bone; and (3) the fracture features in the bone and its replicas.

After each shot, the projectile’s depth of penetration in the gelatine is measured and calculated using images of the trajectory, which is not necessarily rectilinear. The image-based measurement technique takes into account the perspective effects, the parallax as well as the light refraction in order to extrapolate the coordinates of each point of the projectile trajectory in the gelatine block. Because of the uncertainties related to the image reconstruction, penetration depth measurements are reported rounded to the nearest centimetre. After the gelatine is removed, the projectile deformation and bone fractures were also photographically documented.

### Printing parameters and sequence of shots

Table 2 shows an overview of all shots performed on the different biological bones and their corresponding replicas. The table also includes the information on cortical bone thickness and the corresponding printing parameters.

**Table 2:** Information regarding the cortical bone thickness measured on the biological bones, as well as the number of wall layers used in the 3D printed replicas. The table links the target to the projectile type and calibre used for the shooting tests

Biological bone	Mean cortical bone thickness [mm]	Calibre	Projectile
Femur A	4.15	6,35 mm Br.	FMJ
Replica material	# of outer wall layers	Calibre	Projectile
PLA	8 layers of 0.4 mm each	6,35 mm Br.	FMJ
TPU	8 layers of 0.4 mm each	6,35 mm Br.	FMJ
PA6-CF	6 layers of 0.6 mm each	6,35 mm Br.	FMJ
Biological bone	Mean cortical bone thickness [mm]	Calibre	Projectile
Femur B	4.4	.22 LR	LRN
Replica material	# of outer wall layers	Calibre	Projectile
PLA	8 layers of 0.4 mm each	.22 LR	LRN
TPU	8 layers of 0.4 mm each	.22 LR	LRN
PA6-CF	6 layers of 0.6 mm each	.22 LR	LRN
Biological bone	Mean cortical bone thickness [mm]	Calibre	Projectile
Scapula C	1.3	6,35 mm Br.	FMJ
Replica material	# of outer wall layers	Calibre	Projectile
PLA	3 layers of 0.4 mm each	6,35 mm Br.	FMJ
TPU	3 layers of 0.4 mm each	6,35 mm Br.	FMJ
PA6-CF	2 layers of 0.6 mm each	6,35 mm Br.	FMJ
Biological bone	Mean cortical bone thickness [mm]	Calibre	Projectile
Scapula D	2.25	.22 LR	LRN
Replica material	# of outer wall layers	Calibre	Projectile
PLA	5 layers of 0.4 mm each	.22 LR	LRN
TPU	5 layers of 0.4 mm each	.22 LR	LRN
PA6-CF	4 layers of 0.6 mm each	.22 LR	LRN

## Results

### Penetration depth

#### Femur A − 6,35 mm Browning

**Fig. 7:**
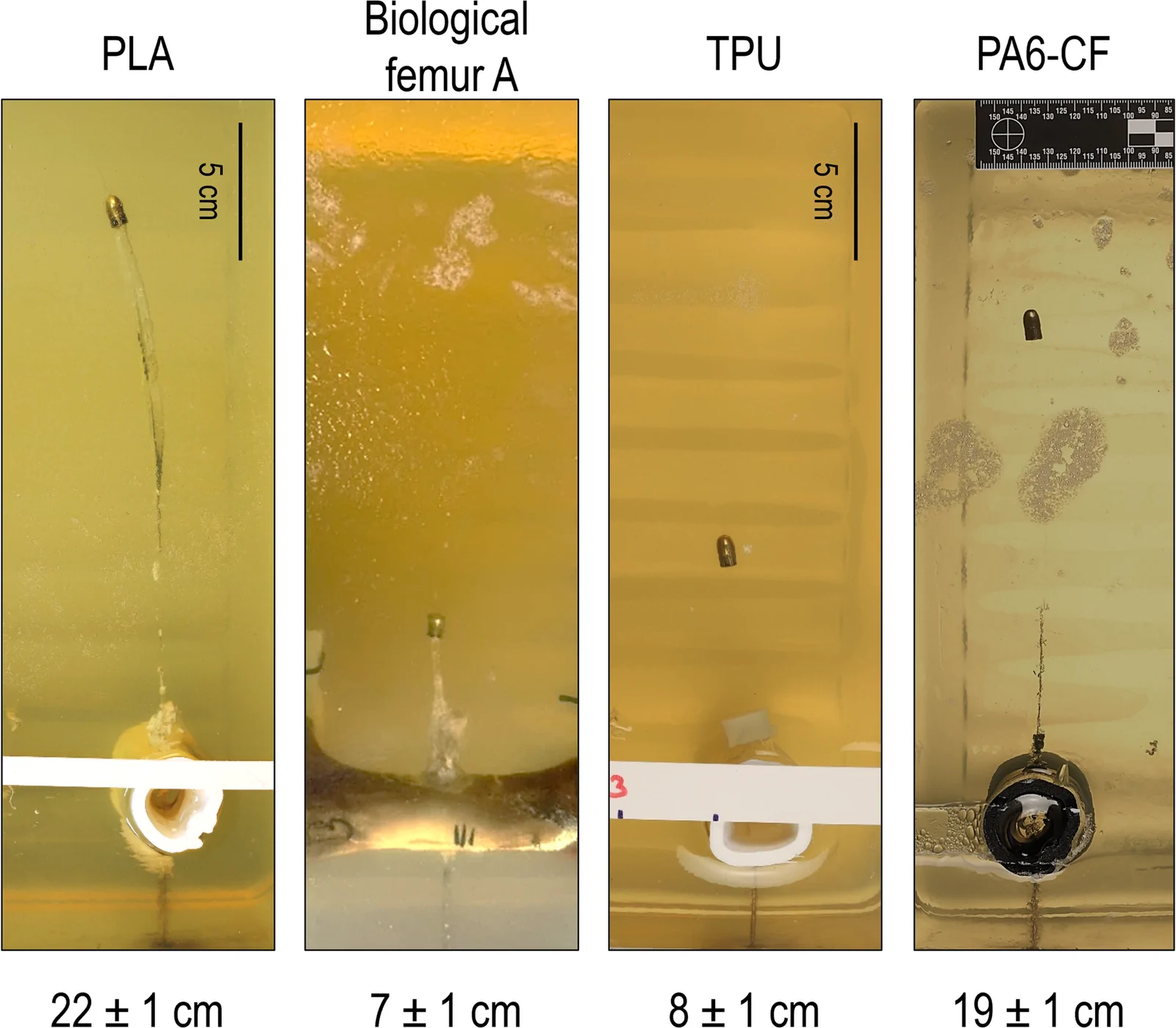
6,35 mm Browning FMJ projectile penetration depth in gelatine of femur A. From left to right: PLA, biological femur A, TPU and PA6-CF. Because of the size difference between the biological bone and the respective replicas (both without epiphysis), the first was placed horizontally into the gelatine, whereas the replicas were placed vertically

The PLA and PA6-CF prototypes show penetration depths of 22 cm and 19 cm, whereas the TPU prototype reaches 8 cm, which is much closer to the 7 cm measure for the reference (Fig. 7).

#### Femur B − .22 Long Rifle

**Fig. 8:**
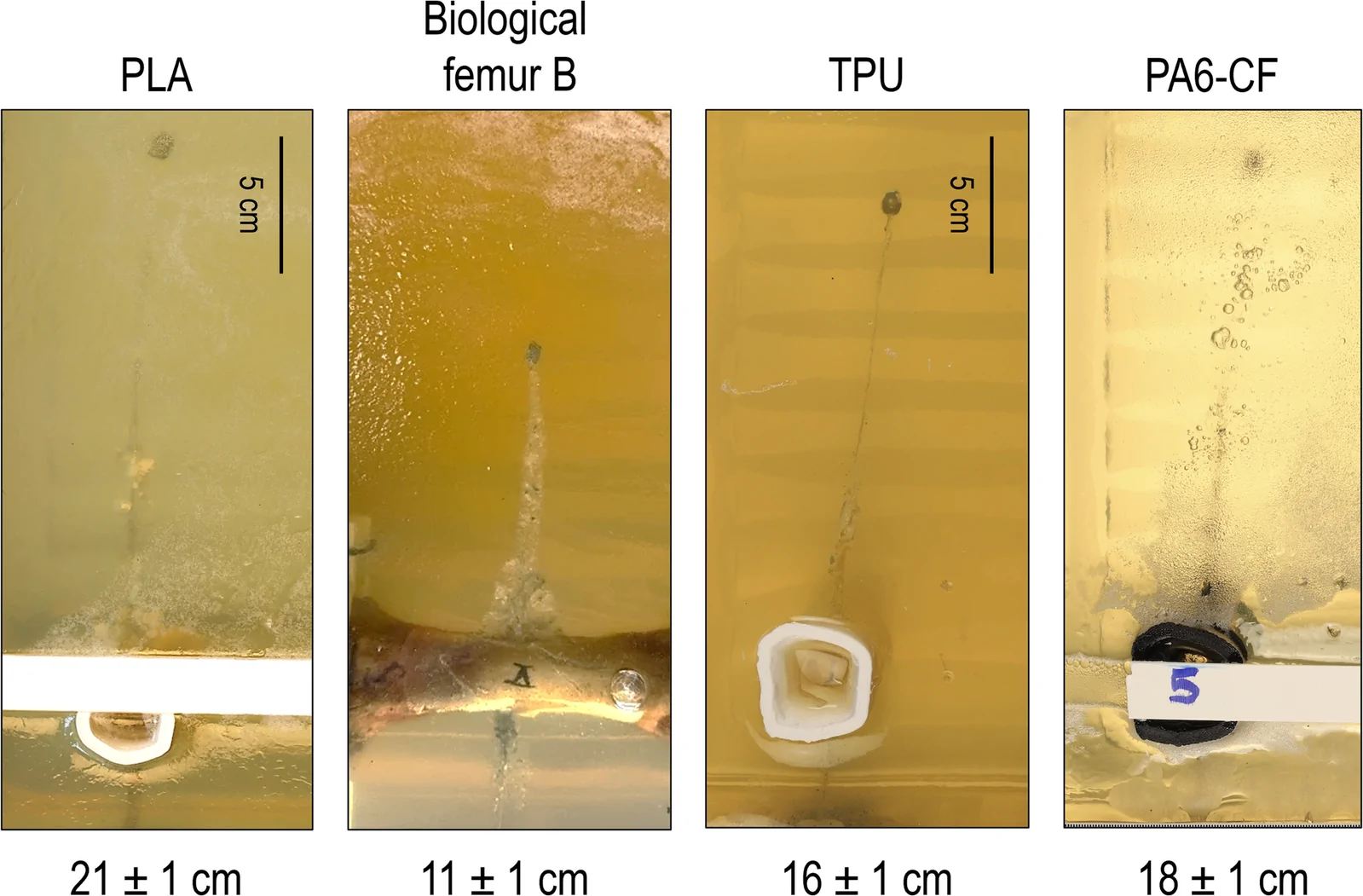
.22 LR projectile penetration depth in gelatine of femur B. From left to right: PLA, biological femur A, TPU and PA6-CF. Because of the size difference between the biological bone and the respective replicas (without both epiphysis), the first was placed horizontally into the gelatine, whereas the replicas were placed vertically

For synthetic Femur B, the three projectiles passed entirely through the PLA, TPU, and PA6-CF bones with penetration depths of 21 cm, 16 cm, and 18 cm, respectively (Fig. 8). The hollow TPU bone is therefore the prototype that achieves the penetration length closest to the reference.

#### Scapula C − 6,35 mm Browning

**Fig. 9:**
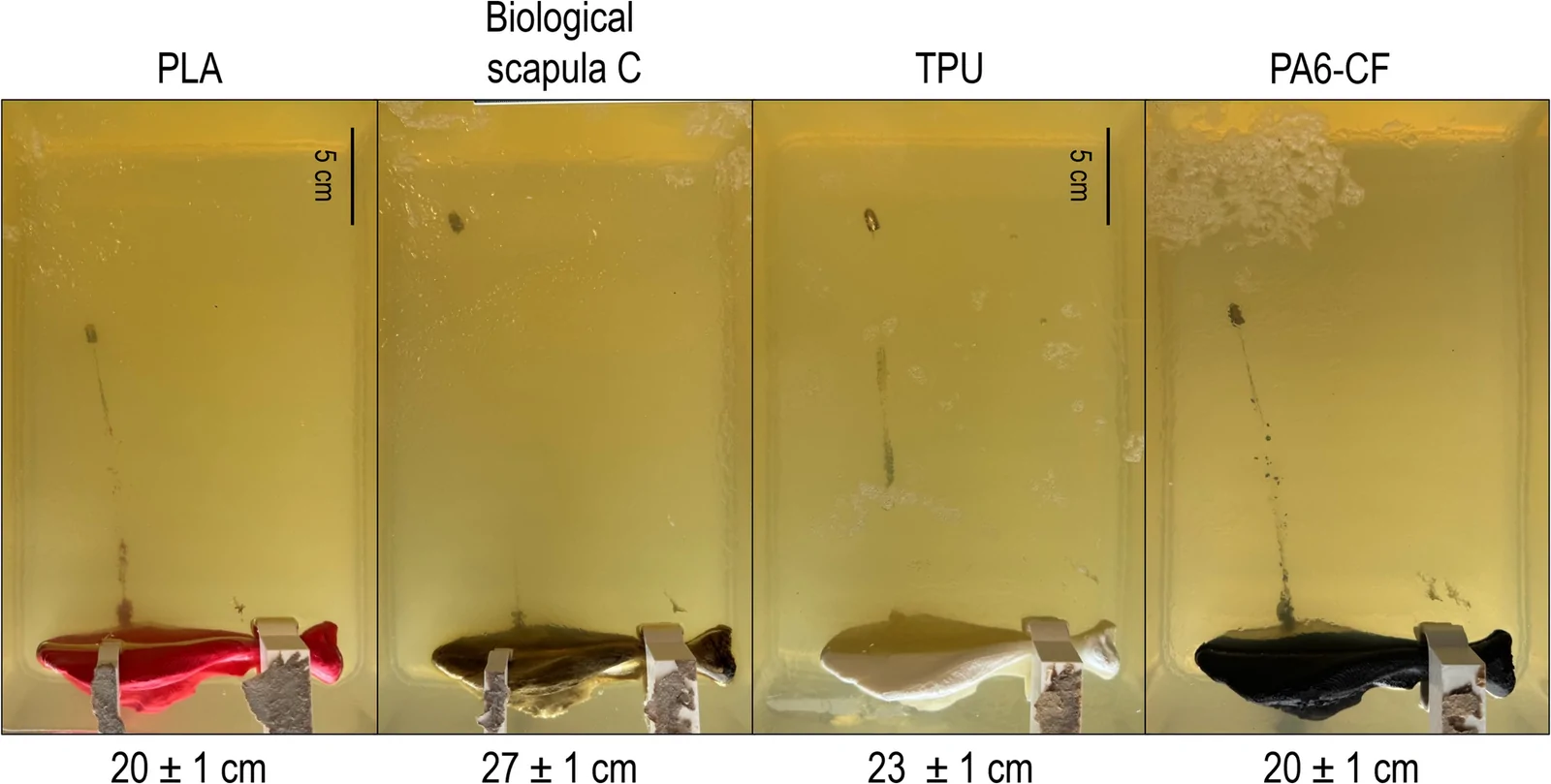
6,35 mm Browning FMJ projectile penetration depth in gelatine of scapula C. From left to right: PLA, biological scapula C, TPU and PA6-CF

Regarding the scapula C impacted by ammunition 6,35 mm Browning, the four bullets passed entirely through the bone and its replicas (Fig. 9). The TPU prototype is closer to the reference in terms of penetration depth, with a projectile reaching 23 cm after the TPU simulant and 27 cm after the biological bone. Greater projectile retention was observed with the PLA and PA6-CF prototypes.

#### Scapula D − .22 Long Rifle

**Fig. 10:**
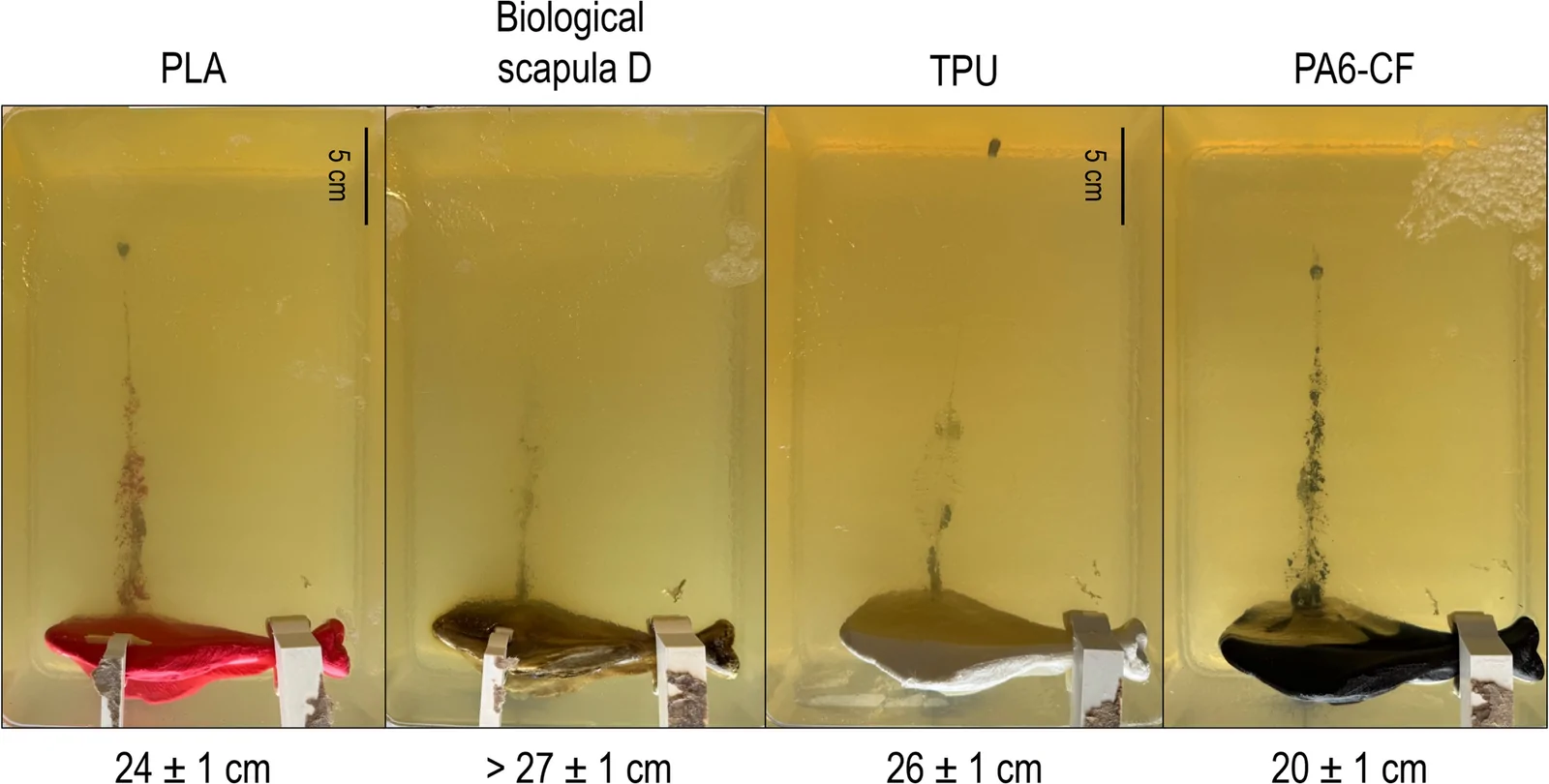
.22 LR projectile penetration depth in gelatine of scapula D. From left to right: PLA, biological scapula D, TPU and PA6-CF

As shown in Fig. 10, the four bullets completely penetrated the scapulas. The reference’s projectile isn’t visible in the gelatine block because it completely passed through the length of the block and lodged in the recovery box behind it. Only its lower penetration limit, approximately 27 cm, can therefore be mentioned. The bullet that perforated the TPU sample nearly perforated the gelatine but stopped at a depth of 26 cm. As with scapula C, greater projectile retention (less penetration depth in gelatine) was observed with the PLA and PA6-CF samples.

### Projectile deformation

#### Femur A − 6,35 mm Browning

**Fig. 11:**
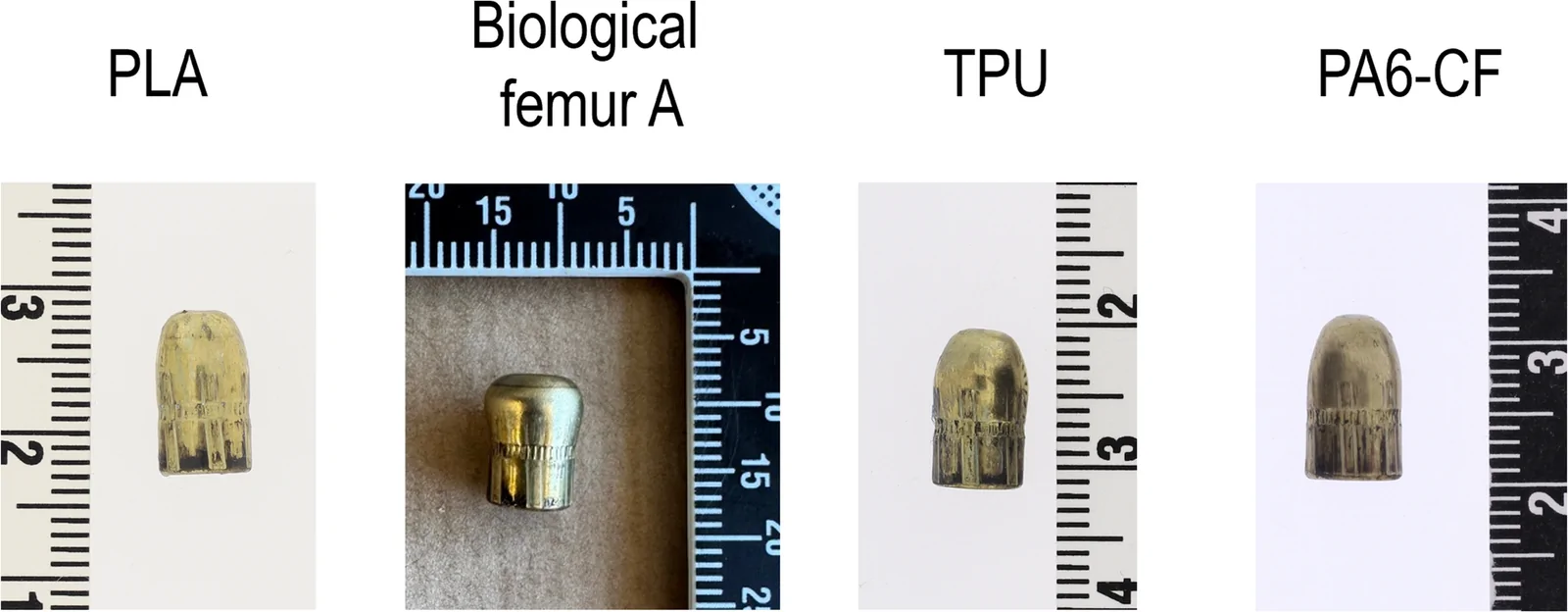
Comparison of the 6,35 mm Browning FMJ projectiles after perforation of the femur A or their replicas. From left to right: PLA, biological femur A, TPU and PA6-CF

All the projectiles that entirely passed through the synthetic bones show similar deformation, less than that which impacted the biological bone (Fig. 11).

#### Femur B − .22 Long Rifle

**Fig. 12:**
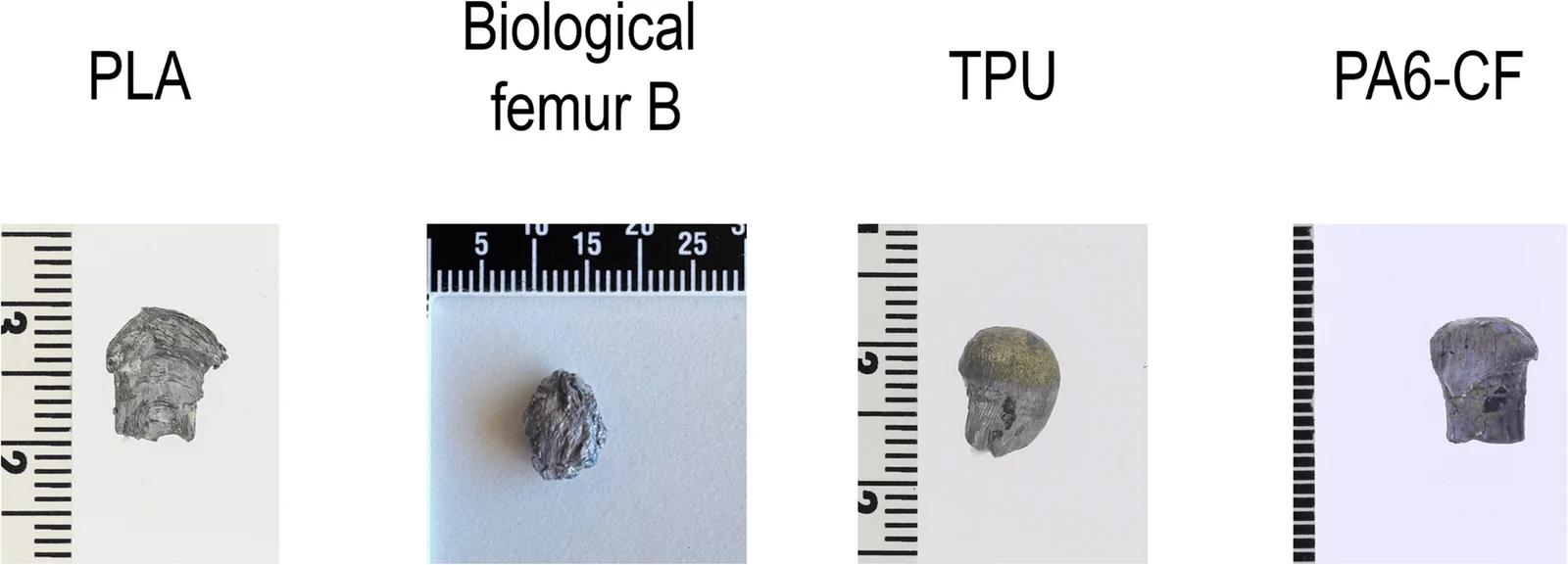
Comparison of the .22 LR projectiles after perforation of the femur B or their replicas. From left to right: PLA, biological femur B, TPU and PA6-CF

As expected, all .22 LR bullets deformed more severely than the 6,35 mm Browning FMJ bullets. The projectile impacting the PLA bone shows a deformation pattern relatively similar to the one that hit the PA6-CF femur, while the one impacting the TPU replica displays rather good preservation of its tip. As a reminder, the latter shows a penetration depth most similar to the reference (Fig. 12).

#### Scapula C − 6,35 mm Browning

**Fig. 13:**
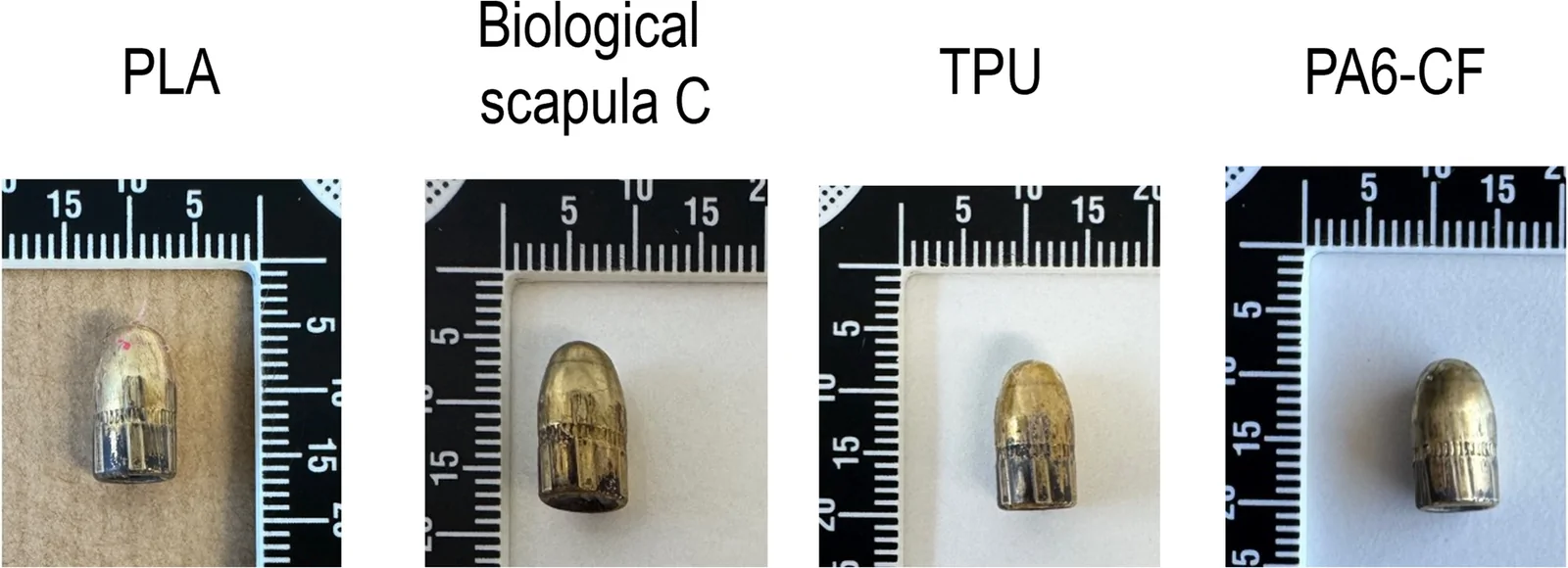
Comparison of the 6,35 mm Browning FMJ projectiles after perforation of the scapula C or their replicas. From left to right: PLA, biological scapula C, TPU and PA6-CF

None of the 6,35 mm bullets that perforated the biological original or the replicas of scapula C deformed noticeably (Fig. 13). It is therefore not possible to perform a meaningful comparison between the bullets.

#### Scapula D − .22 Long Rifle

**Fig. 14:**
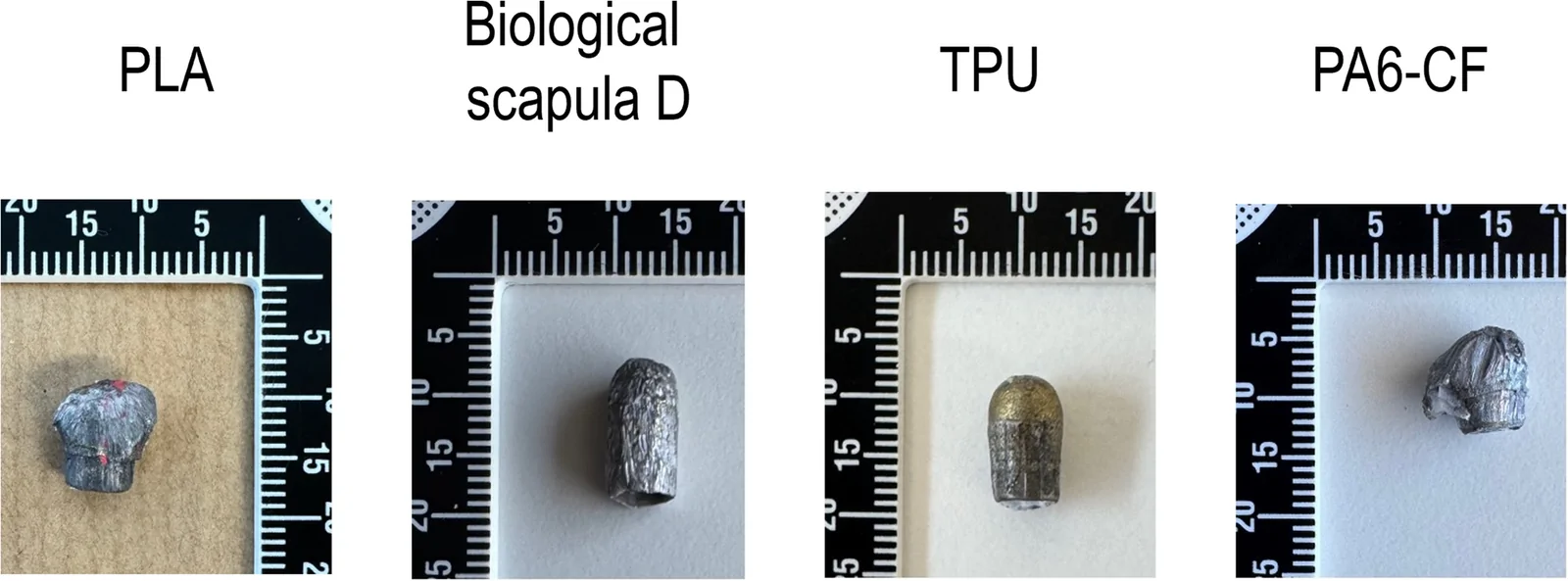
Comparison of the .22 LR projectiles after perforation of the scapula D or their replicas. From left to right: PLA, biological scapula D, TPU and PA6-CF

As noted earlier, projectiles that penetrate the furthest into gelatine generally exhibit less deformation, as evidenced by the projectile that struck the reference and the TPU prototype. In contrast, PLA and PA6-CF projectiles show greater deformation (Fig. 14).

### Fracture characteristics

#### Femur A − 6,35 mm Browning

**Fig. 15:**
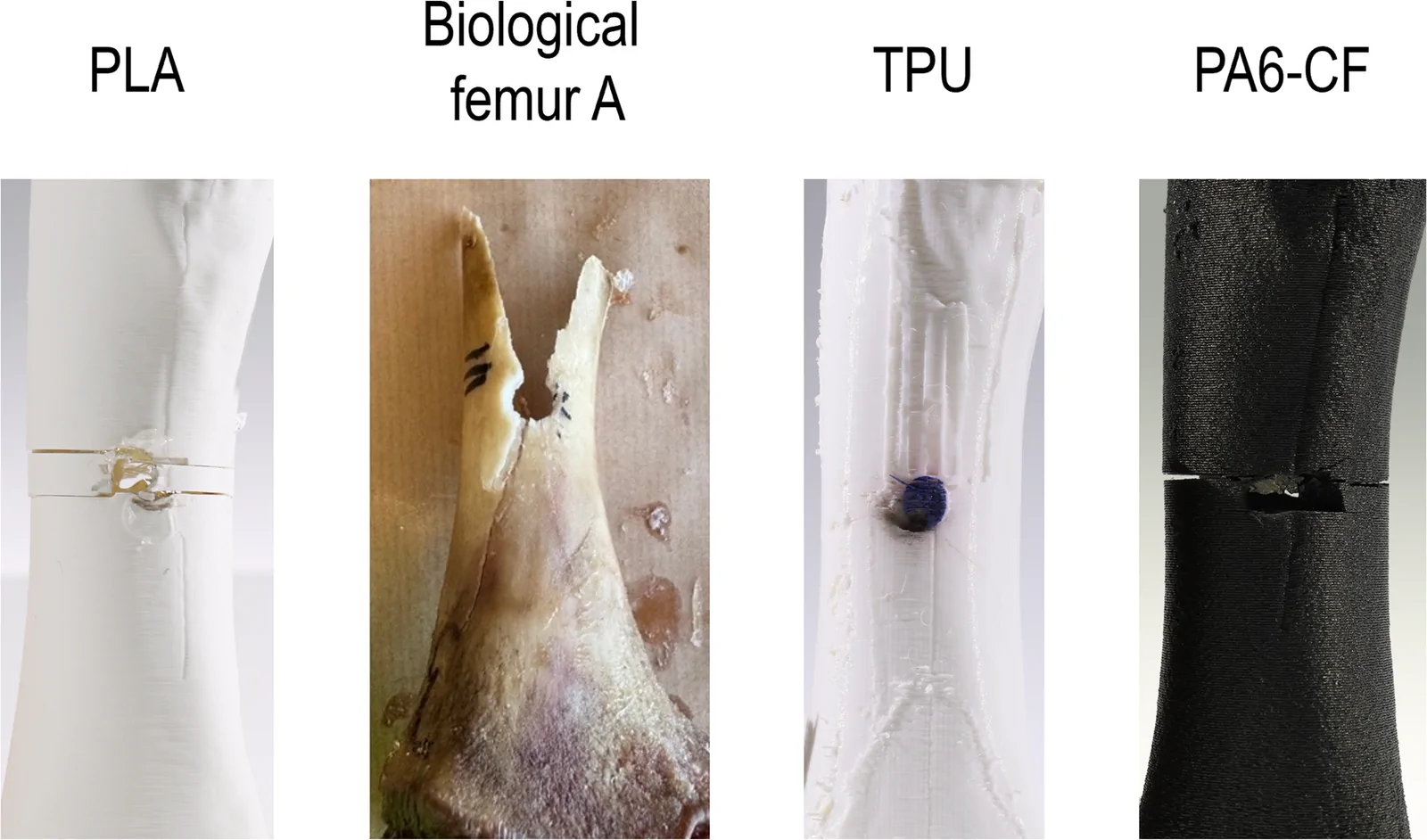
Comparison between the fracture patterns left by the impact of the 6,35 mm Browning FMJ projectile on (from left to right) the PLA replica, the biological femur A, the TPU replica and the PA6-CF replica

Regarding fracture stress of biological and synthetic bone, greater fragmentation was observed on the biological bone than on the synthetic ones. During the analysis of the TPU femur A, no fracture was observed that divides the femur in two. The projectile’s entry hole is visible and well-defined, while the exit hole shows significant fragmentation. In the PA6-CF replica, the pattern consists of a clear fracture line that divides the bone into two distinct pieces. The PLA prototype is the closest to the biological bone in terms of the number of fracture lines, with four in both. The difference is that those in the biological bone are more oblique, radiating out from the entrance orifice, while those on the PLA are parallel to each other, distributed circumferentially around the diameter of the femur (Fig. 15).

#### Femur B − .22 Long Rifle

**Fig. 16:**
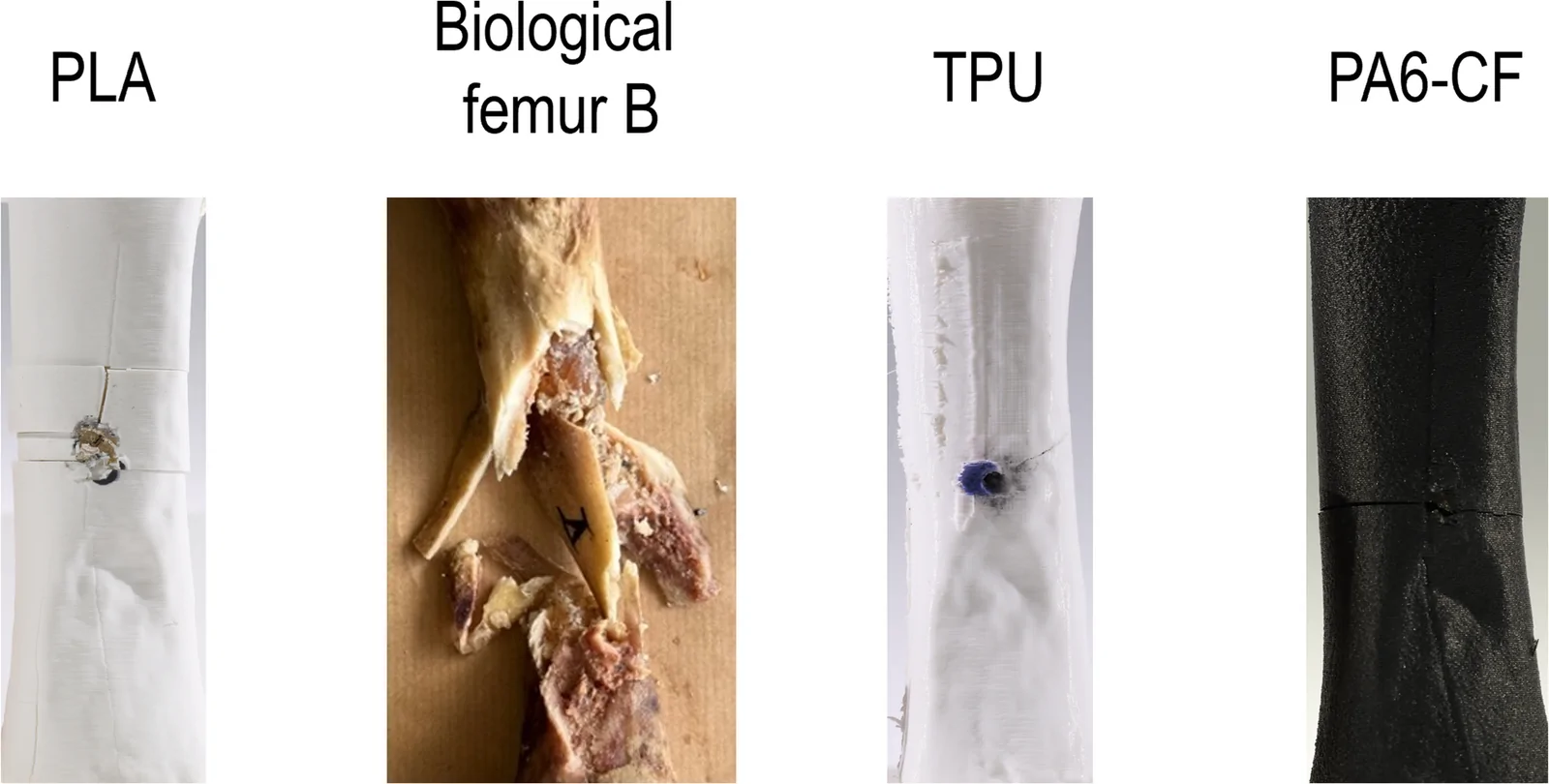
Comparison between the fractures pattern left by the impact of the .22 LR projectile against (from left to right) the PLA replica, the biological femur B, the TPU replica and the PA6-CF replica

As with femur A, the fragmentation of biological bone is greater than that of synthetic bones. Three types of fracture patterns can be observed on the synthetic replicas. Four fractures distributed circumferentially around the diameter of the femur can be counted on the PLA bone, as well as another longitudinal fracture and some fragmentation. The TPU femur, on the other hand, shows a single oblique fracture to the longitudinal axis of the long bone around the entry hole, as well as apparent fragmentation around the exit hole. Finally, as with femur A, the PA6-CF shows a clear fracture line around the diaphysis that divides the bone into two distinct pieces. The synthetic bone showing the most significant fragmentation, and therefore the most similar to the reference, is the PLA bone (Fig. 16).

#### Scapula C − 6,35 mm Browning

**Fig. 17:**
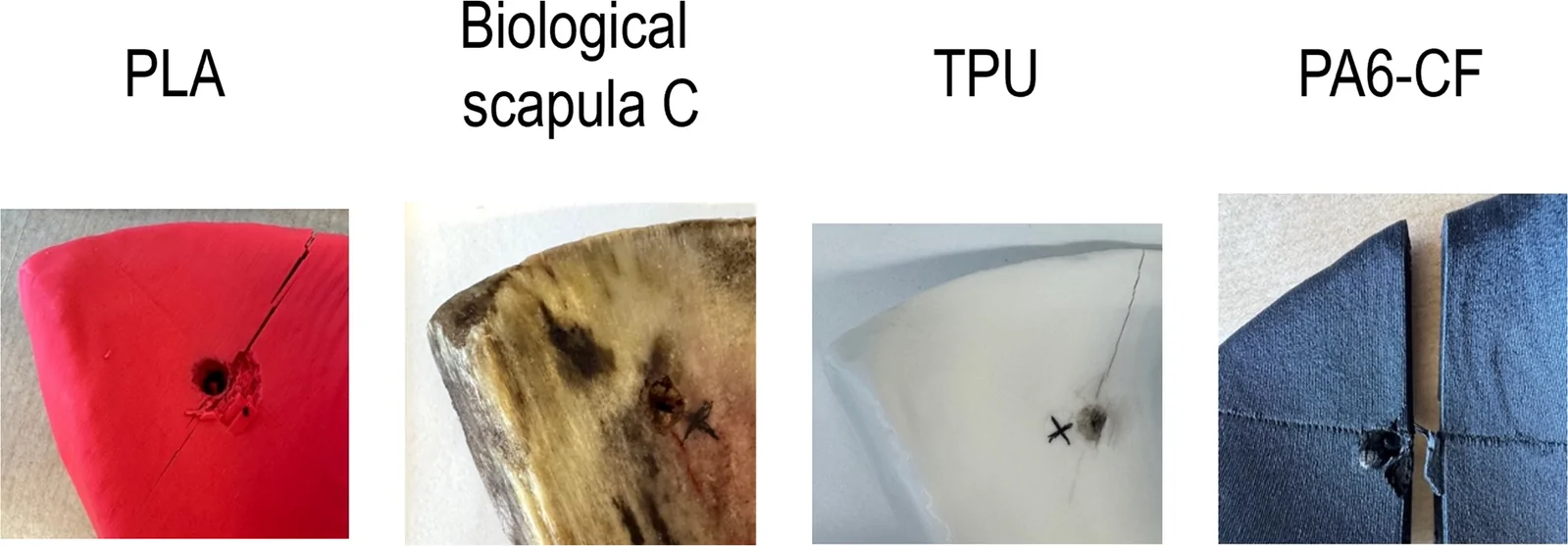
Comparison between the fractures pattern left by the impact of the 6,35 mm Browning FMJ projectile against (from left to right) the PLA replica, the biological scapula C, the TPU replica and the PA6-CF replica

During the fracture stress analysis on scapula C and its synthetic counterparts, no fractures were observed on the reference model, unlike the synthetic scapulas, particularly the PA6-CF, which fractured into two separate pieces. The TPU shows greater similarity to the reference, with only a fine fracture line (Fig. 17).

#### Scapula D − .22 Long Rifle

**Fig. 18:**
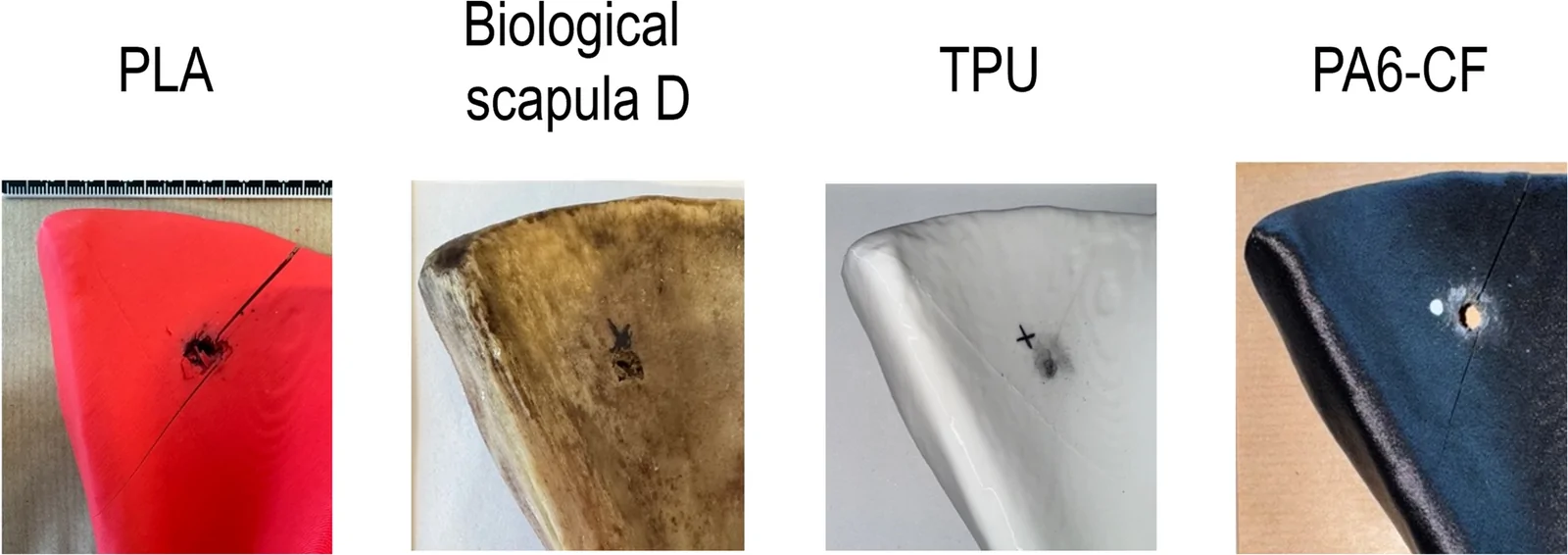
Comparison between the fractures pattern left by the impact of the .22 LR projectile against (from left to right) the PLA replica, the biological scapula C, the TPU replica and the PA6-CF replica

As with the biological scapula D, the reference shows no fracture line, which is closer to the TPU prototype that has only a very fine, barely visible fracture (Fig. 18).

### Summary of results

**Fig. 19:**
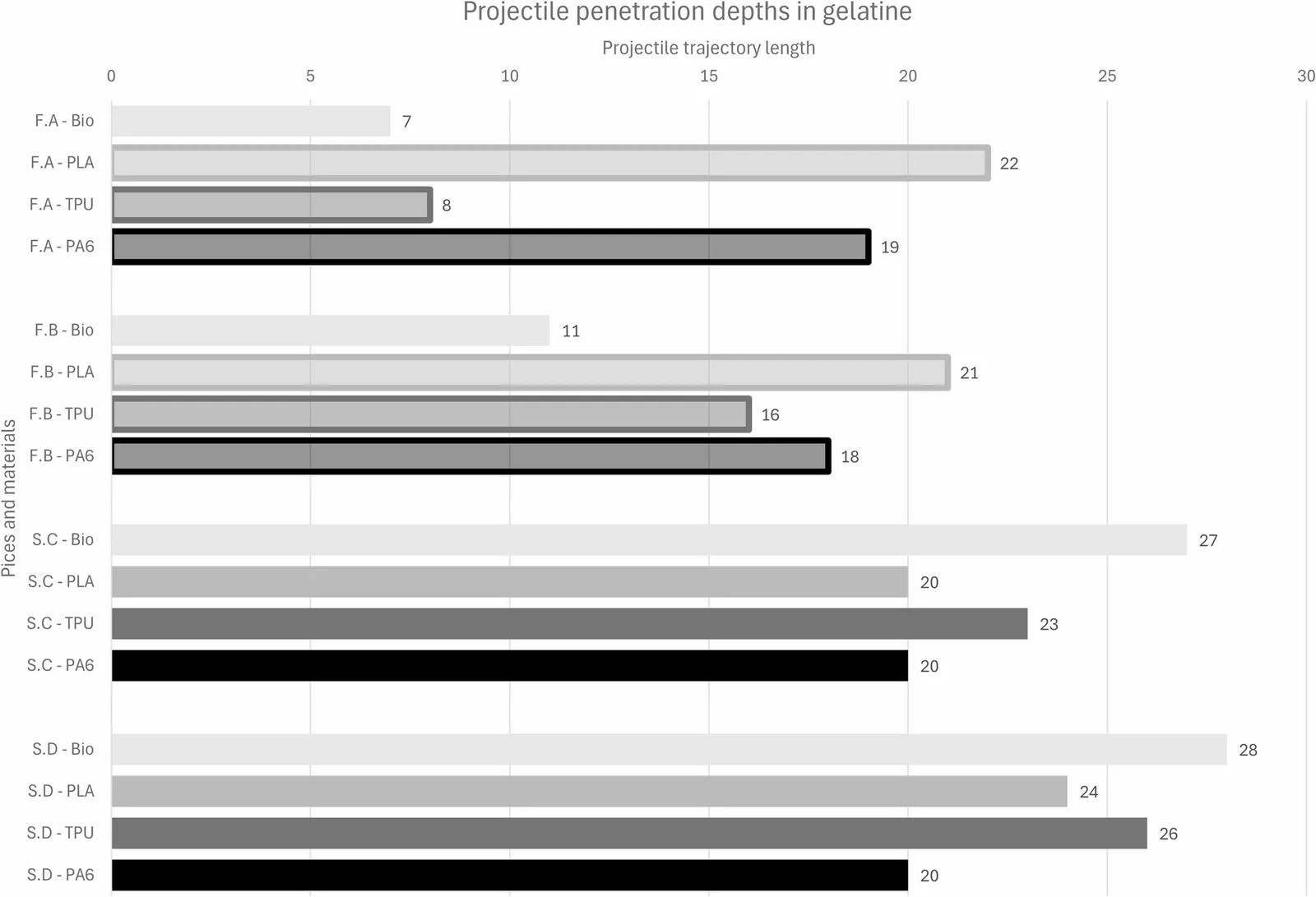
Graph summarizing the penetration depth of all shots on the biological femurs (F.A-Bio and F.B-Bio) and their replicas (F.A-PLA-TPU-PA6 and F.B-PLA-TPU-PA6) as well as the biological scapulas (S.C-Bio and S.D-Bio) and the respective replicas (S.C-PLA-TPU-PA6 and S.D-PLA-TPU-PA6)

Penetration depth might be the key indicator in this study, as it best quantifies projectile behaviour (Fig. 19).

The penetration depths of the projectiles that impacted the PLA and PA6-CF femur replicas are excessive, indicating that this material is less suitable. The TPU femur replicas, on the other hand, show greater similarity to the reference than all other materials. For scapulas, greater similarity to the biological bone is observed with the TPU prototypes for each ammunition type.

## Discussion

### Adopted methodology

The methodology adopted enables a case-specific approach in ballistic reconstructions in cases of gunshot wounds. However, certain conditions must be met. First, the victim’s CT data must be available. If the victim is hospitalised after a shooting incident, there is a good chance that a CT-scan has been performed and that CT data are available. After a fatal outcome, most forensic institutes routinely perform post-mortem CT-scans before the autopsy, which means that CT data will often also be available. One final important limitation is that if bone comminution is severe, it may be difficult to produce a three-dimensional model that is accurate at the impact location.

### 3D printing

The bone representation is restricted to the functionality of the slicer and printer. The infill pattern of the cortical part is set by default to be rectilinear and cannot be adapted to, e.g., bone anisotropy. The cancellous part, on the other hand, has an infill pattern and an infill density corresponding to what cancellous bone is expected to represent, i.e., a honeycomb infill at a density of about 60%. However, it is important to note that the projectiles in this study hit the edges of the infill patterns rather than the « front » pattern, which may affect their interaction. An alternative, which could be further investigated to improve the bone replicas response to the dynamic impact could be the choice of another infill pattern less sensitive to the orientation. About the observation of fracture stresses, it is essential to note that the direction of printing has an influence on some of them. Many fracture lines are parallel to each other in the print direction.

### Limitations and perspectives

As already mentioned, this work should be considered as a pilot study. For each condition tested, only a single 3D-printed specimen was tested (*n* = 1 per condition). This means that the results cannot be generalised beyond the tested specimens at this point. Nevertheless, this study provides an important proof of concept. It demonstrates the practicability of the method, highlights the suitability of the tested materials for shooting reconstruction purposes, and establishes a baseline for follow-up experimentations. Secondly, more research on bone composition and other material properties is needed to improve and guide material research. For preliminary studies used to target satisfactory simulant, it is helpful to use animal bones. However, a comparison with a human reference rather than an animal reference would be preferable to avoid the transposition of the results to humans. Finally, multi-material 3D printers have recently become available and could enable improved representation of the cortical and cancellous regions.

## Conclusion

The objective of this research is to develop a synthetic replica of femurs and scapulas whose interaction with projectiles is similar to that observed in biological bone, serving as a reference. To do this, several ballistics tests were conducted using two calibres, 6,35 mm Browning and .22 Long Rifle, on different materials, including PLA, TPU, and PA6-CF.

Regarding femur replicas, the projectiles that impacted the TPU femurs, filled with 10% gelatine to simulate the bone marrow, show penetration depths closer to those of the reference samples. Still, the projectile deformations and the fractures observed in the bones differed significantly. The PLA and PA6-CF prototypes do not retain the projectiles in the gelatine enough.

The results for TPU scapulas are the most promising. A strong correlation between penetration depth and that in biological bone was observed. Projectile deformation and fracture characteristics are also closer to those of other materials. For the PLA and PA6-CF scapulas, the penetration capacity of the projectiles after impact is lower, unlike the femurs for these materials. The projectiles are therefore more restrained and have shorter penetration depths. The penetration depth is also related to projectile deformation, which is greater than that observed for projectiles impacting biological bones or TPU prototypes. The fractures observed on these synthetic bones are also much more intense.

Finally, because the main aim of this research is to identify the best 3D-printable models for simulating human bones in wound ballistics reconstructions, further ballistics tests could therefore be conducted on different types of bones using these materials to reinforce the reliability of the results.

## Data Availability

No datasets were generated or analysed during the current study.
